# Eosinophilic Esophagitis and Marked Leukocytosis in Alpha-Gal Syndrome: Infection, Allergy, or Hematologic Malignancy?

**DOI:** 10.7759/cureus.116599

**Published:** 2026-09-20

**Authors:** Mohamed Alharami, Christina Kafkis, Omar Alwan, Ziad Suleiman, Hadi Hemaidan, Frhaan M Zahrawi

**Affiliations:** 1 Internal Medicine, Henry Ford Warren Hospital, Warren, USA; 2 Internal Medicine, Prime Healthcare Olympia Fields Hospital, Olympia Fields, USA; 3 Internal Medicine, Hamilton Medical Center, Hamilton, USA; 4 Internal Medicine, Southwest Healthcare Medical Education Consortium, Temecula, USA; 5 Internal Medicine, Herbert Wertheim College of Medicine, Florida International University, Miami, USA

**Keywords:** alpha-gal syndrome, eosinophilic esophagitis (eoe), eosinophilic gastritis (eog), eosinophilic gastrointestinal disorders, hypereosinophilic syndrome

## Abstract

Alpha-gal syndrome (AGS) is a tick-borne immunoglobulin E (IgE)-mediated hypersensitivity reaction to galactose-α-1,3-galactose (alpha-gal) found in mammalian meat products. Eosinophilic gastrointestinal disorders (EGIDs), including eosinophilic esophagitis (EoE), are T helper 2 (Th2)-mediated conditions characterized by eosinophilic infiltration of the gastrointestinal tract. We present a case of a 63-year-old man with known AGS who developed dysphagia, rash, pulmonary infiltrates, gastrointestinal eosinophilic infiltrates, and marked eosinophilia. Bone marrow biopsy revealed marked hypercellularity with myeloid predominance and increased eosinophils and eosinophilic precursors. The patient met the diagnostic criteria for idiopathic hypereosinophilic syndrome (HES) based on persistent peripheral eosinophilia, eosinophilic end-organ damage, and exclusion of secondary and clonal causes. This report describes a rare presentation of AGS meeting the symptomatic and diagnostic criteria of EoE alongside a systemic eosinophilic disorder. The possible shared Th2-mediated pathophysiology of these disorders suggests that AGS could act as an immunologic trigger for these disorders, though further research is needed to establish the association.

## Introduction

Alpha-gal syndrome (AGS) is a tick-borne, delayed hypersensitivity reaction to galactose-α-1,3-galactose (alpha-gal) manifesting as urticaria, anaphylaxis, or gastrointestinal symptoms following the consumption of mammalian meat products [1]. The underlying mechanism is initiated when a tick bite introduces alpha-gal with tick salivary immunomodulator proteins that trigger production of alpha-gal-specific immunoglobulin E (IgE) antibodies. Subsequent consumption of mammalian meat products releases alpha-gal-containing lipoproteins from the digestive system that cross-link with IgE antibodies. In the United States, AGS is most strongly linked to the bite from Amblyomma americanum [1,2]. Recognized risk factors for AGS include ABO blood group status, with blood group B demonstrating protection against sensitization to AGS, male sex, rural residence, and frequent outdoor exposure [1,3,4]. The diagnostic criteria for AGS include positive alpha-gal-specific IgE antibodies, a supporting clinical history, and improvement after cessation of mammalian meat consumption [1].

Eosinophilic gastrointestinal disorders (EGIDs), including eosinophilic esophagitis (EoE), are chronic antigen-driven disorders characterized by eosinophilic infiltration of the gastrointestinal tract. EoE commonly presents with symptoms of dysphagia, odynophagia, and heartburn and is diagnosed based on characteristic symptoms and endoscopic findings, with biopsy showing ≥15 eosinophils/hpf [5].

EoE affects approximately one in 2,000 people and predominates in males, who account for nearly 70% of patients [6]. Additionally, approximately 70% of patients with EoE have current or prior allergic diseases, including AGS [7]. There are limited data examining alpha-gal sensitization as a potential trigger for the development of EGIDs. Both AGS and EoE are CD4+ T helper 2 (Th2)-driven immune responses, each activating interleukins 4, 5, and 13, which in turn promote IgE production and eosinophilic responses [8-10]. In contrast, hypereosinophilic syndrome (HES) is characterized by persistent eosinophilia, with eosinophilic infiltration and end-organ damage. Consensus criteria are met by persistent eosinophilia (>1.5 x109/L) in blood on at least two occasions four weeks apart, or by tissue hypereosinophilia, defined as a bone marrow eosinophil percentage exceeding 20% of all nucleated cells, extensive eosinophil infiltration in the pathologist’s opinion, or marked deposition of eosinophilic granule protein, or by both. In addition to these criteria, there must be organ damage or dysfunction attributable to hypereosinophilia, with other disorders or conditions excluded as the principal cause [11].

We present a rare case of a patient with an established diagnosis of AGS who developed multisystemic eosinophilic infiltrations and had bone marrow findings consistent with idiopathic HES. Our aim is not only to document this uncommon presentation but also to offer clinical reasoning that may help guide further investigation and research.

This article was previously presented as a meeting abstract at the American College of Gastroenterology (ACG) Annual Conference on October 27, 2025 [12].

## Case presentation

A 63-year-old White male patient with a medical history significant only for AGS presented to the hospital in April 2025 with a five-day history of progressive dyspnea, a generalized papular rash (Figures 1A-1D), generalized weakness, edema, low-grade fever, and bilateral pulmonary infiltrates.

**Figure 1:**
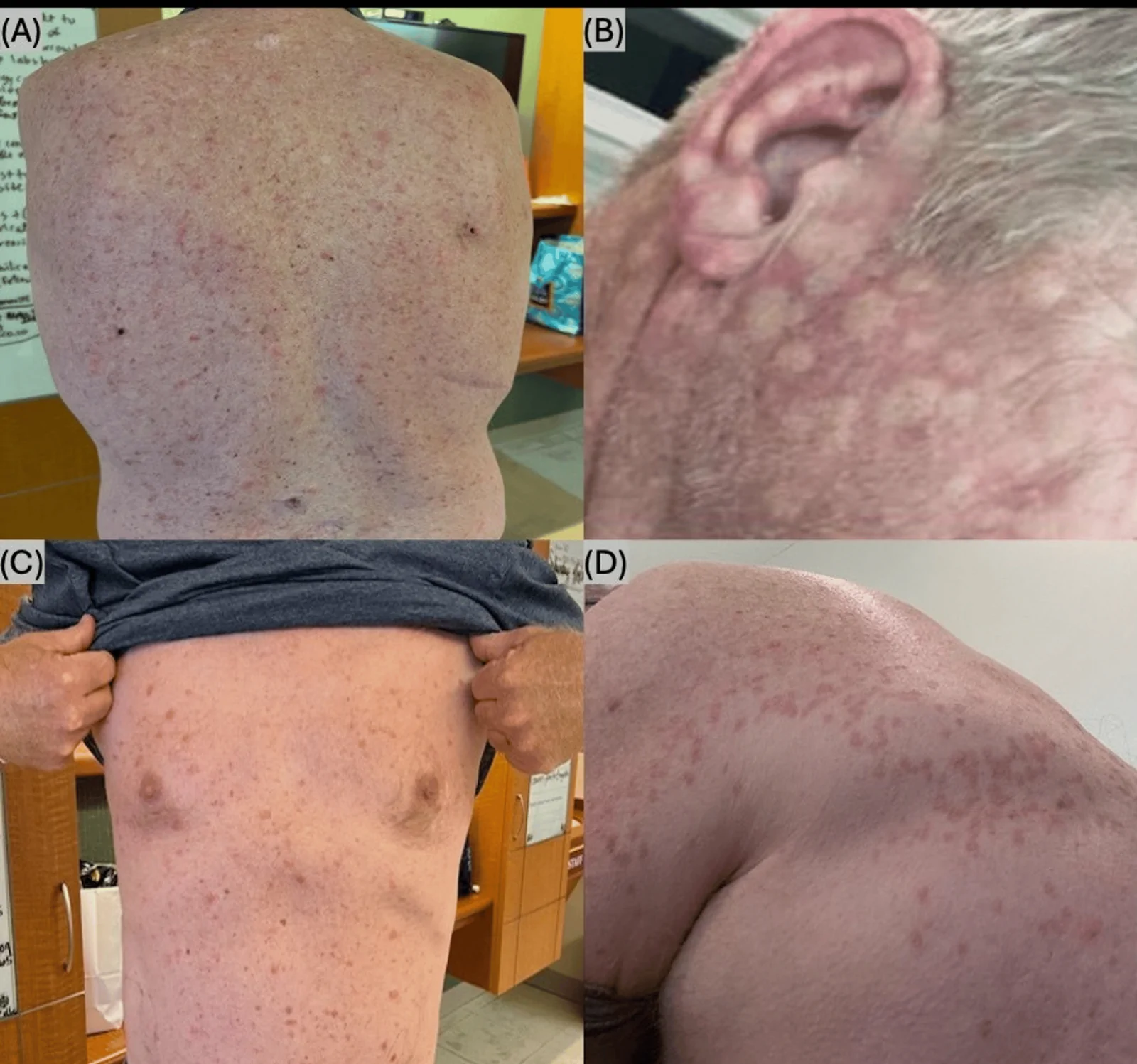
Diffuse cutaneous papular rash on physical examination Erythematous rash involving the (A) back, (B) face and postauricular region, (C) anterior trunk, and (D) shoulder, lower arm, and back. No vesicles, bullae, ulceration, or secretions were noted.

His history was notable for recurrent allergic reactions and generalized rash after consuming mammalian meat, consistent with his previous alpha-gal-related admissions. The patient had been diagnosed with AGS two years before admission after extensive workup for chronic allergic reactions and an episode of anaphylaxis.

Before admission, he was evaluated at an urgent care center and was given one dose of oral ciprofloxacin and doxycycline. Given his high leukocyte count and persistent symptoms, the patient presented to the emergency department for further evaluation. His vital signs were stable and within normal limits. Physical examination revealed a generalized papular rash over the back, anterior trunk, and face (Figures 1A-1D). Pulmonary examination was unremarkable, with no abnormal breath sounds.

Initial laboratory tests revealed rising leukocytosis from 21,600/μL to over 100,000/μL (reference range: 4,000-11,000/μL) with neutrophilia and eosinophilia; the absolute eosinophil count reached a maximum of 42,000/μL (reference range: 0-500/μL) during the hospital course (Table 1).

**Table 1:** Leukocyte and absolute eosinophil count over time D0: urgent care visit; D1: received one dose of ceftriaxone and vancomycin; D2-D6: received doxycycline and cefepime; D6: methylprednisolone initiated for eosinophilic pneumonia; D7: cefepime discontinued and ciprofloxacin initiated due to concern for drug-induced eosinophilia. Leukocyte count: reference range, 4,000-11,000/μL; absolute eosinophil count: reference range, 0-500/μL.

Day (D)	Leukocyte count (×10³/µL)	Absolute eosinophil count (×10³/µL)
0	11.0	0.99
1	21.6	0.40
2	28.6	0.70
3	34.4	1.50
4	39.1	1.90
5	44.3	—
6	45.6	3.40
7	53.6	7.80
8	74.9	—
9	77.6	—
10	102.4	—
12	118.5	42.00
13	120.6	38.20
14	109.2	35.00
25	36.0	17.00
30	19.1	7.50

The patient was initially started on intravenous (IV) vancomycin and IV ceftriaxone in the emergency department, followed by IV cefepime 2 grams every 8 hours during the hospital course.

The initial differential diagnosis included atypical pneumonia, eosinophilic pneumonia, drug-induced eosinophilia (cefepime), and leukemoid reaction. On hospital day 6, IV cefepime 2 grams every 8 hours was discontinued. Per infectious disease recommendation, empiric broad-spectrum antibiotics were continued with oral ciprofloxacin 500 twice daily and oral doxycycline 100 mg twice daily. Given concern for eosinophilic pneumonia, with a rising eosinophil count, IV methylprednisolone 60 mg every 8 hours was initiated per pulmonology recommendation, with consideration of bronchoscopy and biopsy. The patient was monitored with serial laboratory studies and imaging. No treatment-related or procedure-related complications were reported.

Given the patient’s mild dysphagia during the admission, he underwent upper endoscopy, which revealed severe mucosal inflammation of the esophagus (Los Angeles ulcerated grade D esophagitis) and stomach with dense eosinophilic infiltrates, consistent with EoE and eosinophilic gastritis (EG).

Serial laboratory studies demonstrated rising leukocytosis, eosinophilia, and ESR, with a downward-trending CRP from 7.8 to 4 mg/dL (reference range: 0-0.5 mg/dL). A comprehensive infectious, rheumatologic, and malignancy workup was performed. Lactic acid, procalcitonin, and Methicillin-resistant Staphylococcus aureus (MRSA) screen were unremarkable; viral respiratory panel, HIV, Cytomegalovirus (CMV), and malaria blood smear and antigen were negative. Rocky Mountain spotted fever serology, Toxoplasma antibodies, and blood and urine cultures were negative. Autoimmune studies, including antinuclear antibody (ANA), rheumatoid factor, SSA, SSB, anti-Smith, anti-RNP, SCL-70, and anti-double-stranded DNA antibodies, were negative.

The patient underwent multiple computed tomography (CT) scans to exclude an occult infectious source, abscess, or malignancy. CT of the chest revealed stable bilateral posterior basilar infiltrates compared with the initial CT on admission. CT of the abdomen and pelvis showed diverticulosis without diverticulitis, as well as stool burden, and was unremarkable for lymphadenopathy or an infectious source. MRI of the cervical, thoracic, and lumbar spine showed no evidence of discitis, epidural abscess, or osteomyelitis. CT of the right and left hip and CT of the head and soft tissue neck were negative for an infectious source.

Two skin biopsies were performed from the right and left back to rule out cellulitis; they showed interstitial and perivascular inflammation consisting of lymphocytes and eosinophils. The eosinophilic infiltration was more prominent in the left back and was associated with eosinophilic microabscesses, findings characteristic of eosinophilic dermatoses.

Bone marrow biopsy revealed hypercellularity with myeloid predominance and increased eosinophils and eosinophilic precursors. Molecular and cytogenetic studies were negative for PDGFRA, PDGFRB, FGFR1, JAK2, BCR-ABL1, and CBFB rearrangements, which excluded common clonal etiologies. These findings left idiopathic HES as the leading diagnostic consideration.

The patient was discharged on a prolonged course of oral prednisone 60 mg with a gradual taper, proton pump inhibitor therapy, and strict avoidance of mammalian meat products. Close outpatient follow-up with hematology, allergy/immunology, and gastroenterology was arranged. On discharge, the patient underwent alpha-gal testing, which showed a high galactose-α-1,3-galactose IgE level of 1.8 kU/L (reference range: <0.10 kU/L). Corresponding food-specific IgE levels were also elevated, with beef IgE 1.34 kU/L and pork IgE 0.72 kU/L (moderate range: 0.7-3.49 kU/L).

One month after completing the prednisone course, the patient had persistent but improved leukocytosis, with the leukocyte count at 19,100/μL (reference range: 4,000-11,000/μL), with eosinophilic predominance and an improved absolute eosinophil count of 7,755/μL (reference range: 0-500/μL).

## Discussion

This case highlights the complex overlap among AGS, EGIDs, and HES. Although these conditions have distinct clinical features and definitions, they share several immunological features, including a Th2-mediated immune response and eosinophilic inflammation [8-10,13]. Our patient demonstrated a broader eosinophilic phenotype involving the upper gastrointestinal tract, lungs, and skin, with marked eosinophilia in the peripheral blood and bone marrow. We hypothesized that a potential relationship between AGS and EGIDs may be explained by a possible shared Th2-mediated immune response; however, the available literature does not establish that alpha-gal sensitization directly causes a broader eosinophilic phenotype as seen in our case.

The immunologic mechanism underlying alpha-gal sensitization may provide a biological correlation as observed in our patient. Repeated tick exposure following tick bites has been associated with IgE class switching and increased production of alpha-gal IgE [14]. In a study examining skin response to repeated tick bite exposure, individuals with two or more tick bites demonstrated higher alpha-gal IgE levels compared with individuals with one tick bite or healthy controls [15]. Upon histological examination of individuals' skin, an increased type 2 cytokine-producing T-cell response, increased infiltration of basophils and eosinophils, and increased CD4+ T-cells with an increased Th2/Th1 ratio were found.

Emerging evidence suggests that patients with AGS have a robust Th2-mediated immune response to tick protein extract compared with healthy individuals. A recent study of 50 patients with AGS demonstrated that stimulation with tick protein extract induced a clear Th2 cytokine profile and significantly greater T-cell proliferation in patients with AGS compared with healthy controls [10]. Those findings highlight a potential mechanistic link relevant to our patient, who did not present with anaphylaxis but instead demonstrated marked peripheral eosinophilia, as the substantial increase in T-cell proliferation observed in AGS patients could theoretically facilitate the eosinophilic activation and multi-organ infiltration seen in our case. This pathway implies that tick-bite sensitization does more than prime carbohydrate-specific IgE production but may drive generalized mucosal and systemic tissue remodeling through immune hyper-reactivity. Furthermore, additional analysis by Apostolovic et al. examining antibody isotype diversity emphasized that carbohydrate-specific responses interact broadly with Th2-skewed networks [3,10].

Limited literature has examined the association between AGS and EGIDs. A previously reported case described concomitant AGS and EG. That patient presented with primary gastrointestinal complaints and was diagnosed with EG on endoscopic examination. He was subsequently referred to an allergist, who diagnosed AGS [16]. That report suggests that AGS sensitization may coexist with EGIDs. Unlike the previously reported case, our patient presented with multi-system eosinophilic involvement extending beyond the gastrointestinal system, including lung and skin. This suggests that alpha-gal sensitization could be linked to a broader spectrum of eosinophilic disease than previously recognized, potentially modulated by individual genetic predispositions, environmental co-triggers, or overlapping hyper-reactivity syndromes that escalate localized response into systemic eosinophilia.

Additionally, one prospective case-control study examined alpha-gal sensitization as a risk factor for EoE [17]. This study analyzed 100 subjects: 50 EoE cases and 50 non-EoE controls. Twenty-two subjects were sensitized, carrying alpha-gal-specific IgE. Sensitization rates did not differ between the EoE cases and non-EoE controls. The investigators were therefore unable to support their initial hypothesis. The investigators acknowledged the need for further investigation into a potential link between alpha-gal sensitization and EoE development. However, these findings do not exclude a role for AGS as a plausible driver of EoE. While alpha-gal is not a universal driver of standard EoE, it may trigger disease in a highly susceptible patient subset, which may cause more severe, systemic presentations. This is further supported by broader investigations into food allergy intersections with EoE, suggesting diverse antigenic exposure and atypical gastrointestinal presentations can amplify mucosal inflammation [9].

More comprehensive clinical and translational studies are needed to clarify the relationship between AGS and primary eosinophilic disorders and to explore whether AGS may unmask or exacerbate the underlying eosinophilic gastrointestinal pathology. One avenue of research would be to determine whether prior Th2 immune activation, such as that seen in AGS, influences the future development of EGIDs. Given the limitations of a single case report, causality cannot be established. However, the temporal association, possible shared Th2 immune pathway, and multisystem eosinophilic involvement suggest a possible relationship between AGS and increased susceptibility to EGIDs and HES.

## Conclusions

This case discusses a rare co-existence of EGIDs and idiopathic HES in a patient with known AGS, illustrating that tick-induced alpha-gal sensitization can manifest beyond the traditional cutaneous and gastrointestinal symptoms. This case demonstrated systemic involvement, hypereosinophilia, and bone marrow hypercellularity, which would not be expected in traditional AGS cases. The presence of multi-organ tissue infiltration and marked leukocytosis, as seen in this case, requires clinicians to maintain a broad differential diagnosis that includes evaluating for HES.
